# Proning related bilateral anterior ischaemic optic neuropathy in a patient with COVID-19 related acute respiratory distress syndrome

**DOI:** 10.1186/s12886-021-02028-9

**Published:** 2021-07-13

**Authors:** Kirsty Michelle Clarke, Vivi Riga, Amy-lee Shirodkar, Joel Meyer

**Affiliations:** 1 Department of Ophthalmology, Moorfields Eye Hospital NHS Foundation Trust, London, UK; 2 Department of Intensive Care Medicine, Guy’s and St Thomas’ NHS Foundation Trust, London, UK; 3 Department of Ophthalmology, Guy’s and St Thomas’ NHS Foundation Trust, London, UK

**Keywords:** Compression ischaemic optic neuropathy, Critical care, Intensive care, Proning, Pneumonitis, SARS-CoV- 2, Visual impairment, Vision loss

## Abstract

**Background:**

Non-arteritic ischaemic optic neuropathy (NAION) is a rare but harmful complication of prone positioning. Prone mechanical ventilation is a therapeutic strategy which has been used extensively during the COVID-19 pandemic to treat acutely hypoxemic patients with COVID-19 related acute respiratory distress syndrome (ARDS). Though a small number of cases of unilateral NAION have been reported in patients testing positive for the SARS-CoV-2 virus, we describe what is to our knowledge, the first reported case of bilateral NAION occurring in a patient proned extensively for the treatment of COVID-19 related ARDS. We consider the potential aetiological factors leading to NAION after prone mechanical ventilation in patients with COVID-19 and suggest strategies to protect against its development.

**Case presentation:**

: We report a case of severe, irreversible, visual impairment secondary to bilateral anterior ION in a fifty-five-year-old male who underwent eight episodes of prone mechanical ventilation to treat COVID-19 related ARDS. Once weaned from his sedation he reported bilateral painless vision loss, and bedside ophthalmological assessment identified a reduced visual acuity of 3/30 unaided in the left eye and counting fingers in the right. Dilated indirect ophthalmoscopy revealed inferotemporal optic disc oedema with splinter haemorrhages in the right eye and mild disc oedema, temporal pallor, and nerve fibre layer haemorrhages inferiorly in the left eye. Humphrey visual field 24 − 2 testing confirmed a severely constricted visual field with macular sparing on the right and depressed inferonasal vision with preserved peripheral vision on the left eye. OCT disc imaging shortly after diagnosis revealed bilateral disc swelling and flame haemorrhages in the right eye.

**Conclusions:**

NAION is a devastating, but preventable complication of prone positioning, which may pose significant risk of vision loss in patients with COVID-19 related ARDS.

## Background

NAION is a cause of permanent vision loss, which occurs when the optic nerve’s blood supply is interrupted, leading to irreversible nerve fibre atrophy[1]. Anterior NAION occurs due to infarction of the posterior ciliary arteries supplying the optic disc, and posterior NAION occurs due to infarction of the pial capillary plexus which supplies the retrolaminar optic nerve[1]. The retrolaminar optic nerve is particularly susceptible to hypoperfusion injury, as the pial vessels are incapable of autoregulatory pressure control, rendering them vulnerable to watershed infarction[1].

Clinically, NAION presents as sudden, painless irreversible vision loss, which typically occurs upon waking[2].Early ophthalmic assessment is central to the diagnosis of NAION in patients with COVID-19 related ARDS, as their ability to report visual loss is limited by the use of tracheal intubation and deep sedation in the context of critical illness[3]. Anterior NAION can be diagnosed by the presence of a swollen or less commonly hyperaemic, haemorrhagic optic discs on fundoscopy[1]. Early indicators of posterior NAION are more subtle, including blurred vision, reduced colour vision, and a relative afferent pupillary defect (RAPD)[2]. In their later stages, both anterior and posterior NAION present with optic disc pallor due to advanced nerve fibre degeneration[1].

COVID-19 related ARDS is a dysregulated inflammatory response to SARS-CoV-2 within the lung Parenchyma[4]. Prone mechanical ventilation has proven mortality benefit in patients with ARDS[5], and has been used extensively to treat COVID19 related ARDS. However, prone mechanical ventilation is an established risk factor for NAION development[3]. Prone positioning was first linked to NAION by reports of postoperative vision loss in patients undergoing prone spinal surgery, for which the incidence of perioperative vision loss is believed to be as high as 0.2 %[6]. Notably, NAION has been reported in perioperative patients undergoing prone positioning for an average of 497 +/- 180 min, a significantly shorter duration than that used for critically ill COVID-19 patients[6, 7].

The mechanism of NAION in patients undergoing prone mechanical ventilation to treat COVID-19 related ARDS is multifactorial. Prone positioning is thought to alter ocular haemodynamics, leading to persistently raised intraocular pressure (IOP) and reduced optic nerve perfusion[8]. In critically unwell patients with severe COVID-19 related ARDS, optic nerve hypoperfusion may also be exacerbated by the methods used to manage multi-organ failure, such as renal replacement therapy, prolonged immobility and deep sedation[3]. Importantly, COVID-19 infection is an independent risk factor for microvascular disease, and recent evidence has highlighted COVID-19 related hypoxia and thrombophilia as biologically plausible mechanisms for NAION development[9].

While cases of unilateral NAION have been described in patients testing positive for the SARS-CoV-2 virus, here we discuss what is to our knowledge the first reported case of proning associated bilateral NAION in a patient with COVID19 related ARDS[9–12].

## Case presentation

**Fig. 1:**
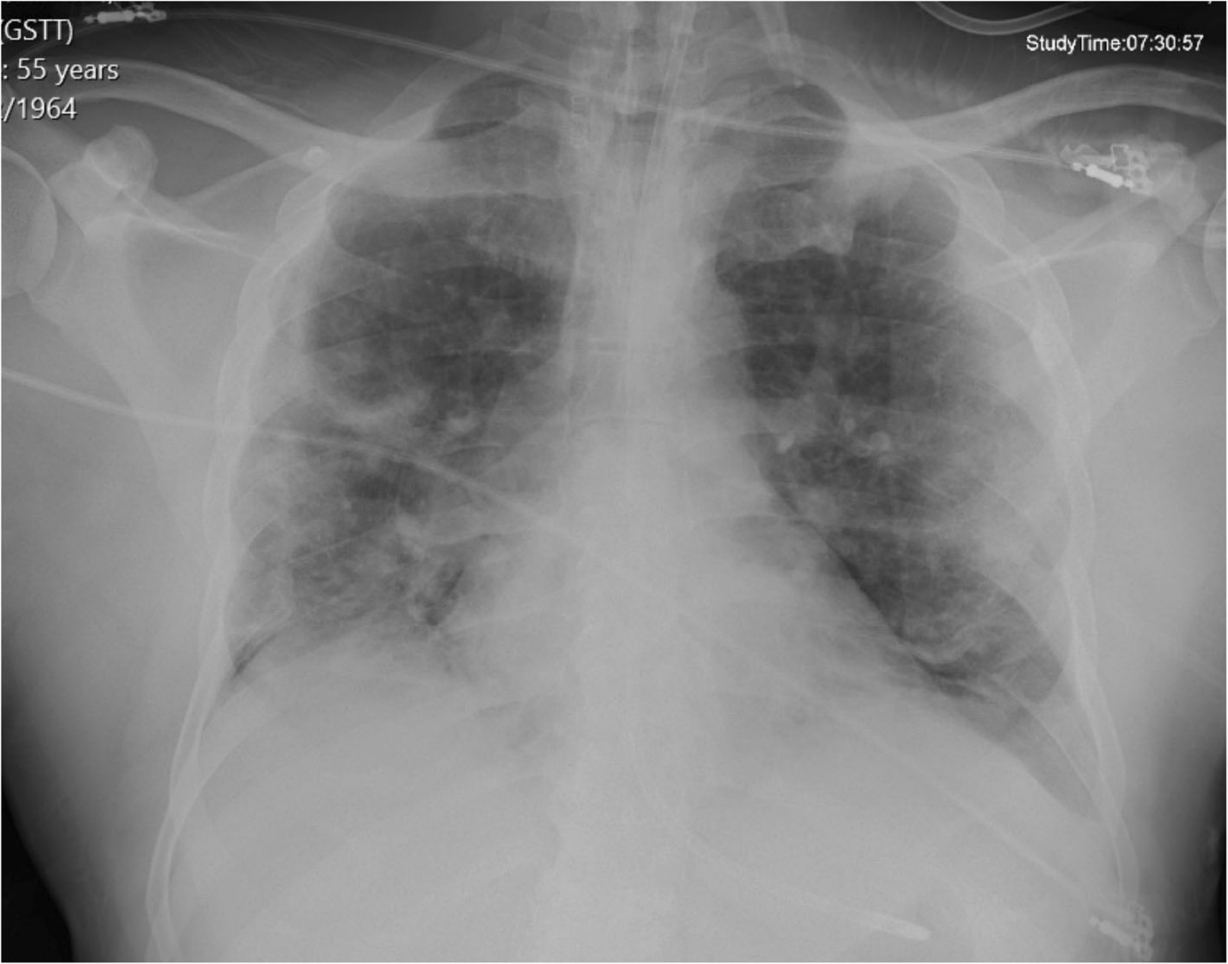
AP erect chest radiograph Legend - AP erect chest radiograph showing bilateral air space opacifications predominantly in the mid and lower zones

A fifty-five-year-old afro-Caribbean male (CP) presented to a central London emergency department in April 2020 with worsening dyspnoea, a dry cough and anosmia. He was an ex-smoker, and his past medical history included hypercholesterolemia and hypertension. He had no visual complaints or ocular history. On examination, he had a raised temperature of 39.7’C, blood pressure of 166/90mmHg and respiratory rate of 40 bpm. When placed on 5 L per minute of supplemental oxygen via a nasal cannula, he could not maintain oxygen saturations above 92 %. Therefore, he was sedated, intubated, mechanically ventilated, and admitted to intensive care. Admission blood tests showed raised inflammatory markers; CRP (90 mg/l), ferritin (421ng/ml), and leukopenia (0.7 × 109/l). His triglycerides were 1.56mmol/l, and his blood glucose was raised, although his Hba1c was within range (4.6 %). Clotting studies revealed an INR of 1.1, fibrinogen of 6.8 g/L and d-dimer of 1.26 mg/L. Two consecutive nasopharyngeal swabs confirmed SARS-CoV-2 infection and a chest radiograph showed bilateral heterogeneous opacification of upper-middle and lower zones, consistent with a diagnosis of COVID19 pneumonitis (Fig. 1).

CP remained in the ICU for nine weeks with hypoxemic respiratory failure necessitating prolonged sedation and eight episodes of prone mechanical ventilation, each lasting between 16 and 22 h. He experienced multi-organ failure, requiring treatment with vasopressors and four weeks of renal replacement therapy. His recovery was complicated gastrointestinal bleeding, with a Hb drop below 70 g/L, requiring blood transfusion. A CT abdomen revealed intramural haematoma within the terminal ileum, which was complicated by evolving long-segmental ischaemia and multiple splenic infarcts. Following cessation of episodic proning and when neurological assessment was feasible it became apparent that CP had right arm weakness. Brain imaging excluded an acute intracranial event, and an MRI scan of the right shoulder revealed replacement of the subscapularis muscle with organising haematoma confirming a diagnosis of proning-related right brachial plexopathy.

**Fig. 2:**
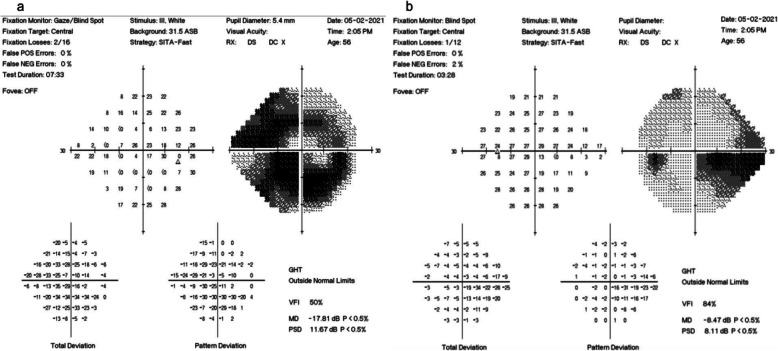
Humphrey visual field 24 − 2 testing. Legend- **a** Humphrey visual field 24 − 2 testing of the right eye showing severely constricted visual field with an island of macular sparing. The test is reliable enough for interpretation with an acceptable number of false positives (0 %) and false negatives (0 %). **b** Humphrey visual field 24-2 testing of the left eye showing depressed inferonasal vision with preserved peripheral vision. The defect is altudinal as it largely respects the horizontal meridian. The test is reliable enough for interpretation with an acceptable number of false positives (0%) and false negatives (2%).

**Fig. 3:**
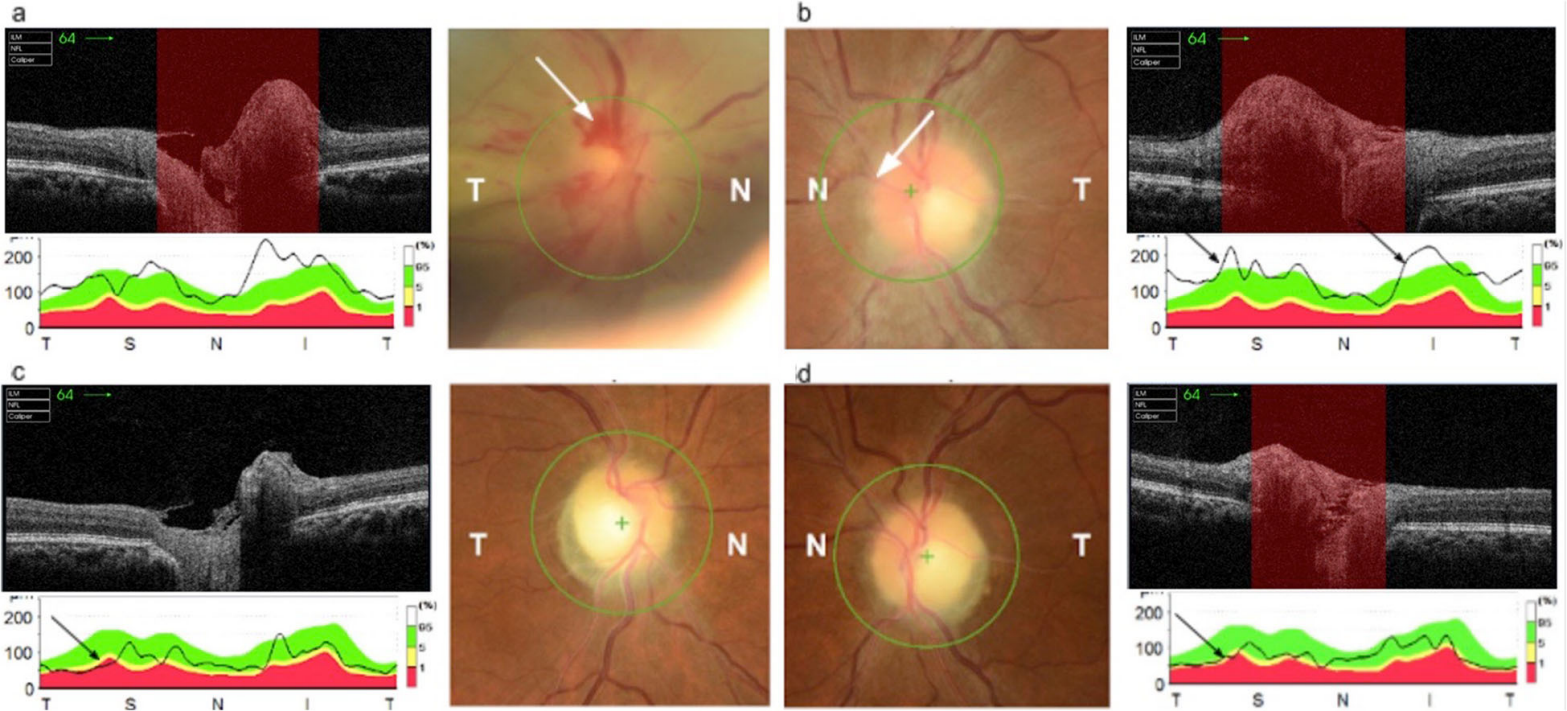
OCT disc imaging. **a** OCT disc of the right eye at the time of diagnosis showing flame haemorrhages (black arrow) and disc swelling evidenced by blurring of the disc margins. Disc swelling is also evident on the OCT cross section of the disc (white arrow). **b** OCT disc of the left eye at the time of diagnosis showing disc swelling evidenced by blurring of the disc margins (black arrow) and thickening of the retinal nerve fibre layer (black arrow). **c** OCT disc of the right eye 2 months after initial diagnosis, showing disc pallor when compared to previous OCT of the right eye and atrophy of the retinal nerve fibre layer as depicted by the RNFL graph (black arrow). **d** OCT disc of the left eye 2 months after initial diagnosis, showing disc pallor when compared to previous OCT of the left eye and atrophy of the retinal nerve fibre layer as depicted by the RNFL graph (black arrow)

After cessation of sedation, CP reported new-onset profound bilateral vision loss, more pronounced on the left. A bedside ophthalmological assessment revealed a reduced visual acuity (VA) of 3/30 unaided in the left eye and counting fingers in the right. His IOPs were normal, 10mmHg bilaterally, and a RAPD was present in the right eye. Dilated indirect ophthalmoscopy revealed bilateral features of NAION including sectoral (inferotemporal) optic disc oedema with evidence of splinter haemorrhages in the right eye and mild disc oedema, temporal pallor, and nerve fibre layer haemorrhages inferiorly in the left. Five weeks later, Humphrey visual field 24 − 2 testing confirmed a severely constricted visual field with macular sparing on the right and depressed inferonasal vision with preserved peripheral vision on the left (Fig. 2). OCT disc imaging of both eyes shortly after diagnosis revealed bilateral disc swelling and flame haemorrhages in the right eye (Fig. 3). Repeat OCT discs at two months follow-up showed disc pallor and thinning of the retinal nerve fibre layer in both eyes. CP’s VA showed no improvement, and he was referred to the low vision clinic to provide advice regarding driving and registering for low vision support.

## Discussion and conclusion

Prone positioning is a life-saving measure for many critically unwell patients with COVID-19. However, intensive care staff should be aware of the ocular complications associated with its use, especially as the irreversible vision loss caused by NAION can be devastating for survivors and their families. With awareness of the risk factors which predispose patients to NAION, such as uncontrolled hypertension, sleep apnoea, diabetes mellitus and hypercholesterolaemia, measures can be taken to protect against its development, without interfering with lifesaving treatment[3]. The following discussion first explains how NAION was diagnosed in this case, before highlighting the key aetiological factors contributing to its development. Finally, we recommend measures to protect against NAION in critically unwell patients with COVID-19 where prone positioning may be unavoidable.

Diagnosis of NAION cannot be made without a timely ophthalmic assessment, including VA, IOPs, pupillary responses, confrontational visual fields and fundoscopy[2]. As in this case, bedside fundoscopy can reveal nerve layer haemorrhages and edematous optic discs, which are highly suggestive of anterior NAION in a patient with cardiovascular risk factors[1]. Other features of optic nerve damage, such as reduced VA, colour vision loss and RAPD should also be present[1, 2]. A dilated fundus examination is essential to exclude treatable causes of painless vision loss such as retinal detachment and vitreous haemorrhage[2]. In unilateral NAION examination of the unaffected can also identify patients with “discs at risk”, smaller, crowded optic discs predisposed to ION[2].

An important differential for NAION is central retinal artery occlusion which presents with profound vision loss and a characteristic cherry red spot on fundoscopy[8]. Central retinal artery occlusion has been reported in patients proned perioperatively who experience fluctuations in mean arterial pressure. This occurs as blood supply to the optic nerve is auto-regulated by changes in terminal arteriolar resistance, a mechanism which breaks down at the extremes of mean arterial pressure[13]. In this case, CP’s mean arterial pressure was kept between strict limits of 70-100mmHg, further excluding this diagnosis.

In addition to ophthalmological assessment, brain imaging and a neurological assessment should be conducted in any patient with acute vision loss and cardiovascular risk factors, to exclude a cerebral vascular incident[2]A biochemical blood panel including CRP or ESR is also necessary to exclude giant cell arteritis[2]. Once stable, patients with suspected NAION should undergo urgent ophthalmology referral for slit-lamp examination, OCT imaging, formal visual acuity and fields. These measures help determine the extent of a patient’s vision loss so that they can receive advice regarding driving and qualification for low vision support[2]. More detailed testing can also help elucidate the cause of NAION, allowing targeted risk factor modification to protect any remaining vision.

Prone positioning is an established risk factor for NAION as it can cause alterations in IOP which precipitate nerve hypoperfusion. IOP fluctuations can occur due to external compression of the orbit, via a process resembling orbital compartment syndrome, or indirectly due to increased central venous pressure (CVP[8]. Raised IOP occurring without external pressure to the orbit can occur as no valves are present in the orbital veins, leaving the eye especially vulnerable to positional changes, as raised CVP can translate directly into raised IOP[14].

Knowledge of how prone positioning can lead to NAION may aid its prevention, for example a 10° reverse Trendelenburg position can reduce IOP compared to the neutral or 5° position[3]. Venous return is especially likely to be compromised if the head remains to one side for extended periods, or malpositioning leads to direct pressure on the abdomen obstructing venous return to the heart. Attempts to position patients with the head level or above the heart, aiming to maintain blood pressure within 20 % of baseline, may significantly reduce the risk of ION development[13].

Prone positioning is a key risk factor for the development of NAION in patients with COVID-19 related ARDS. However, multiple other risk factors may have compounded CP’s risk of NAION. For example, life-saving treatments used in conjunction with prone positioning to treat multi-organ failure in critically unwell patients, such as sedation, vasopressors, and anaesthesia, increase the risk of NAION by overwhelming the autoregulatory mechanisms of optic nerve perfusion[3]. These treatments may be unavoidable, but screening for vision loss in patients who have been proned can prevent delayed diagnosis, and late referral for vision support. Blood tests for lipids, Hba1c and blood pressure checks are also important to identify cardiovascular risk factors, which can be controlled to significantly reduce the risk of NAION.

Notably, recent evidence has highlighted the biological plausibility of COVID-19 related hypoxemia and hypercoagulability as independent risk factors for NAION[9–12]. As NAION is a disease of circulatory insufficiency, it follows that hypoxemia caused by COVID-19 related ARDS may precipitate NAION in prone patients already at risk of hypoperfusion injury[9]. The relationship between COVID-19, hypercoagulability and microvascular disease is still being explored, but is likely to relate to a combination of endothelial injury caused by direct viral invasion and a prothrombotic state triggered by immune-mediated hyperinflammation, viral ACE2 receptor binding and in-situ immunothrombosis[15]. COVID-19 related immunothrombosis may be particularly concerning for the microvascular inflammation and micorembolic disease seen in NAION, and there is reason to believe that careful anticoagulation should play a central role in the management of patients undergoing prone ventilation to treat COVID-19 related ARDS[9–12].

In conclusion ION is a devastating, but preventable complication of proning, which should be considered in COVID-19 patients treated with prone mechanical ventilation.

## Data Availability

Not applicable.

## Abbreviations

ARDS: Acute respiratory distress syndrome
ION: Ischaemic optic neuropathy
IOP: intraocular pressure
RAPD: Relative afferent pupillary defect
VA: Visual Acuity
